# Supplementary material for: Tailoring of Dissimilar Friction Stir Lap Welding of Aluminum and Titanium
Source: Materials (Basel). 2022 Nov 26;15(23):8418. doi: 10.3390/ma15238418 (PMC9738075; doi:10.3390/ma15238418)
Supplement: Supplementary file 1 [file materials-15-08418-s001.zip › materials-2000303-supplementary.pptx]

## Slide 1
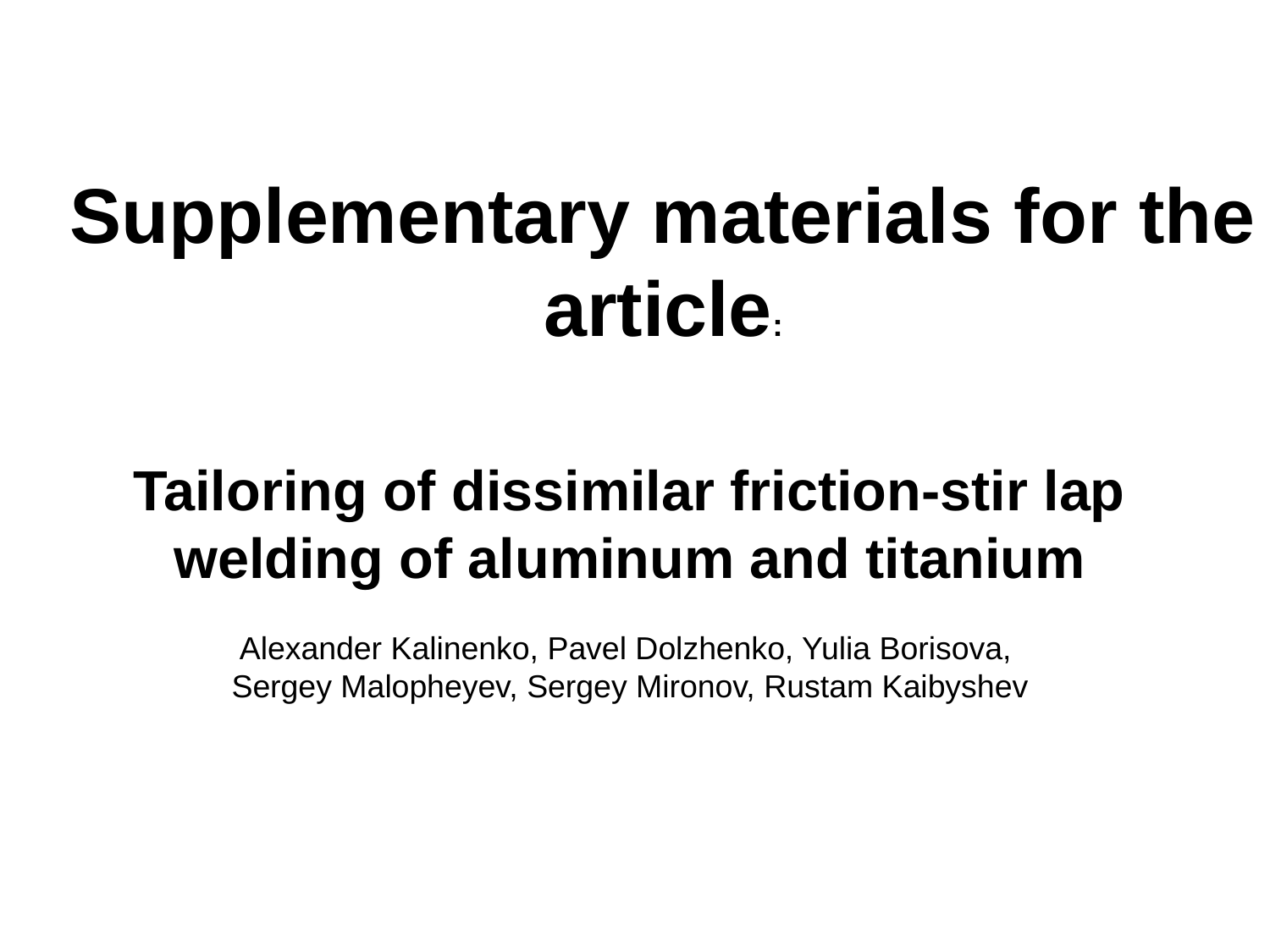

Supplementary materials for the article:
Tailoring of dissimilar friction-stir lap welding of aluminum and titanium
Alexander Kalinenko, Pavel Dolzhenko, Yulia Borisova,
Sergey Malopheyev, Sergey Mironov, Rustam Kaibyshev

## Slide 2
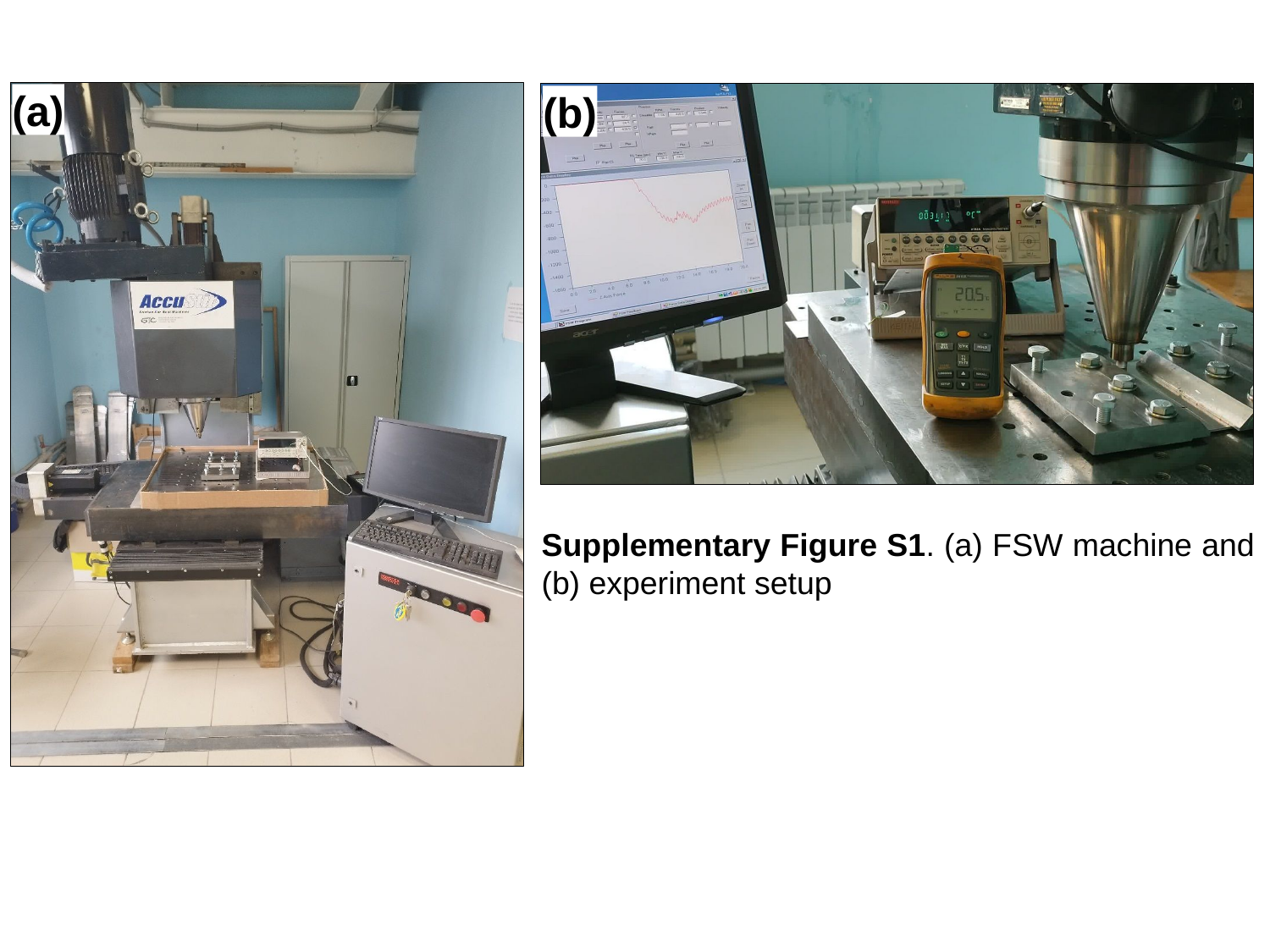

(a)
(b)
Supplementary Figure S1. (a) FSW machine and (b) experiment setup

## Slide 3
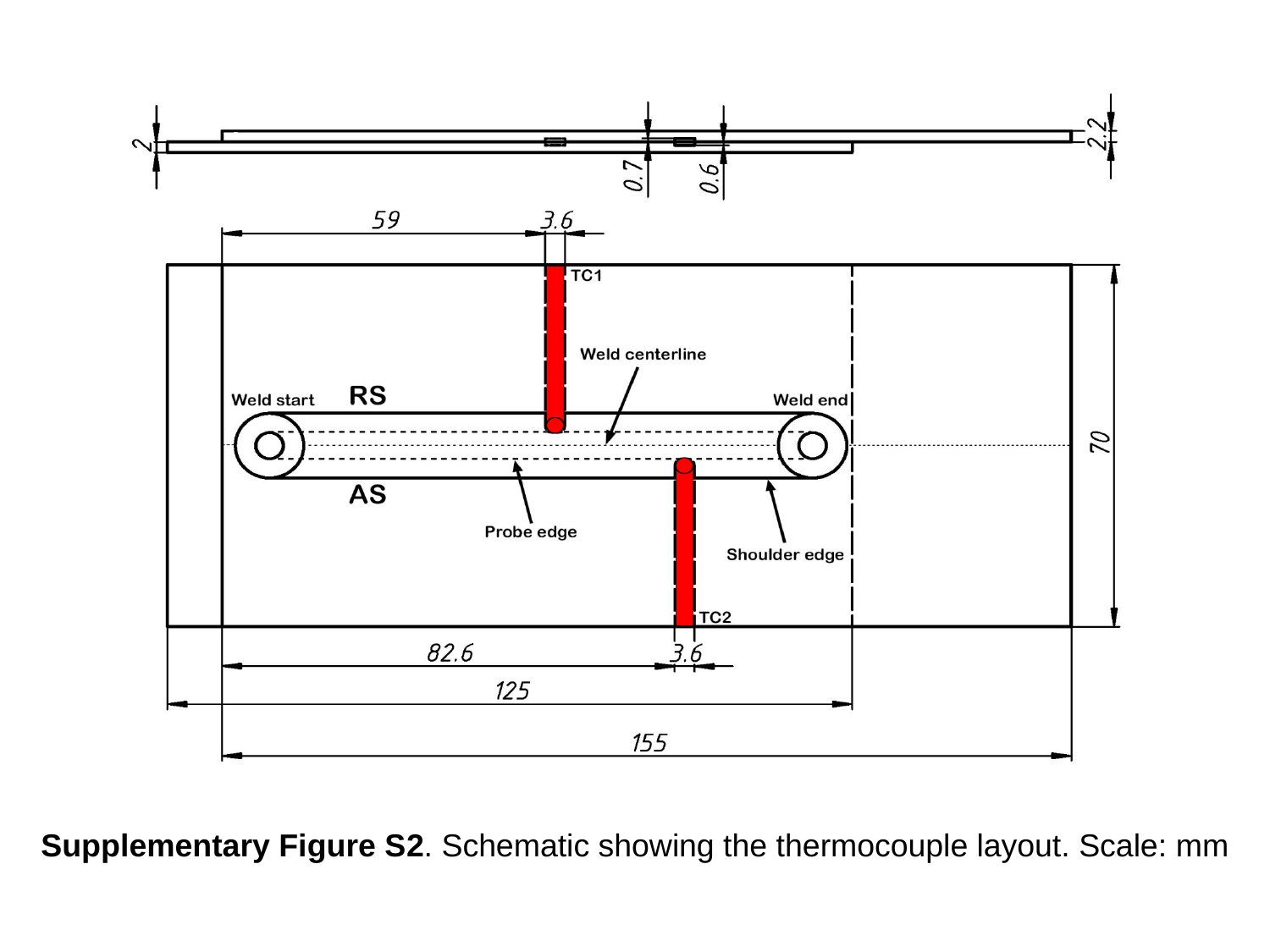

Supplementary Figure S2. Schematic showing the thermocouple layout. Scale: mm

## Slide 4
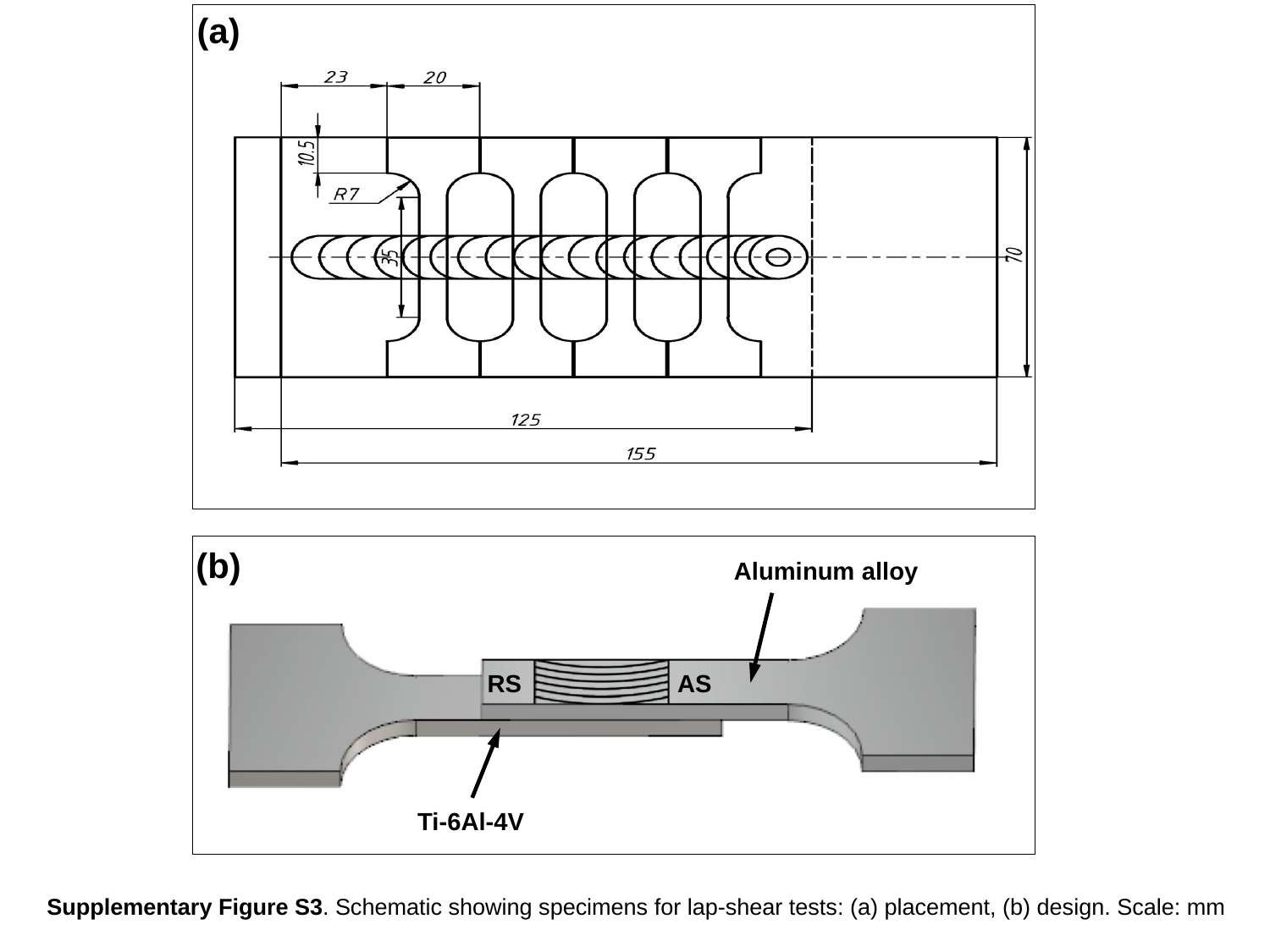

(a)
Aluminum alloy
Ti-6Al-4V
(b)
RS
AS
Supplementary Figure S3. Schematic showing specimens for lap-shear tests: (a) placement, (b) design. Scale: mm

## Slide 5
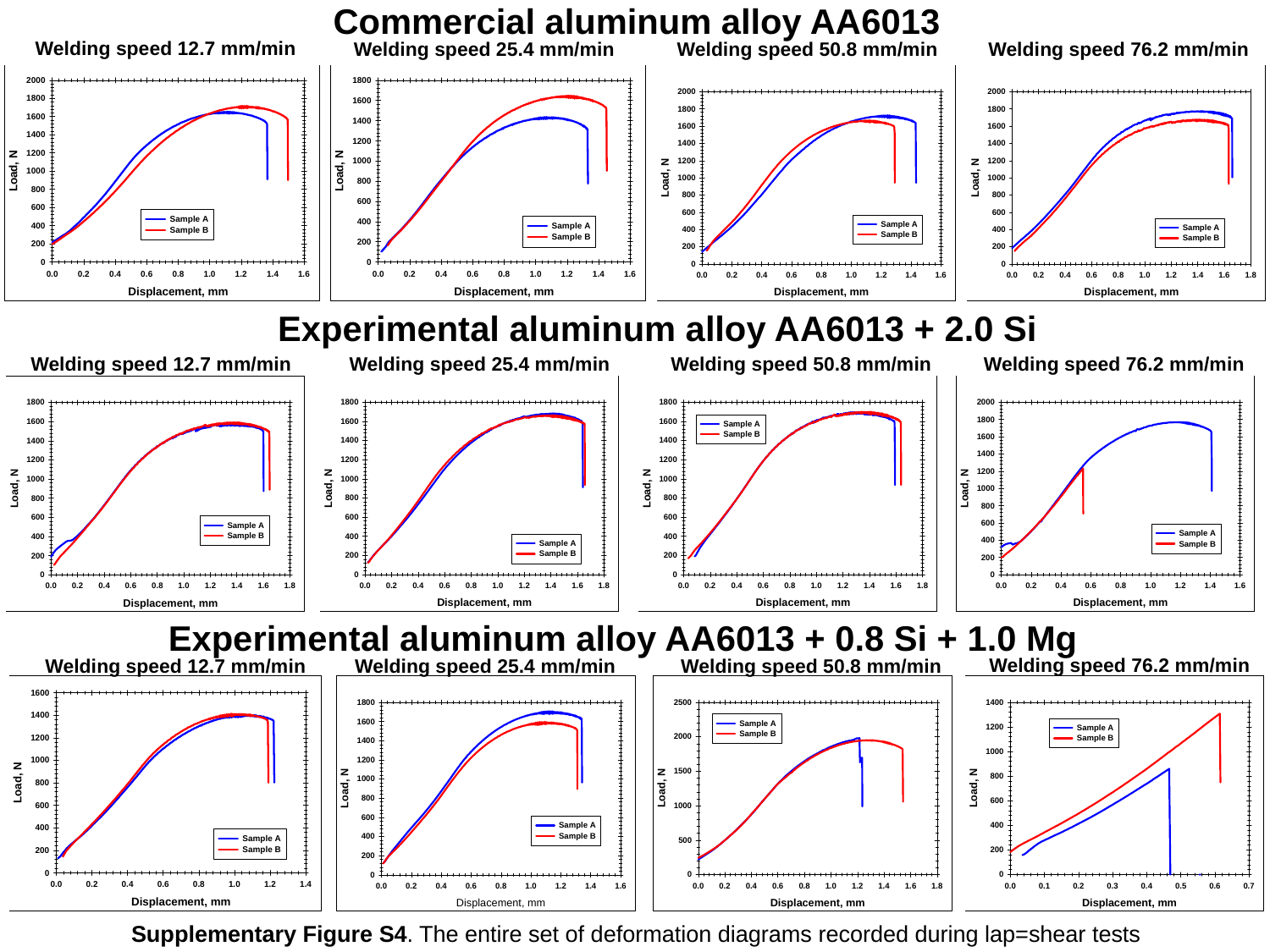

Commercial aluminum alloy AA6013
Welding speed 12.7 mm/min
Welding speed 25.4 mm/min
Welding speed 50.8 mm/min
Welding speed 76.2 mm/min
Experimental aluminum alloy AA6013 + 2.0 Si
Welding speed 12.7 mm/min
Welding speed 25.4 mm/min
Welding speed 50.8 mm/min
Welding speed 76.2 mm/min
Experimental aluminum alloy AA6013 + 0.8 Si + 1.0 Mg
Welding speed 76.2 mm/min
Welding speed 25.4 mm/min
Welding speed 50.8 mm/min
Welding speed 12.7 mm/min
Supplementary Figure S4. The entire set of deformation diagrams recorded during lap=shear tests
